# Urinary Cell-Free DNA in Bladder Cancer Detection

**DOI:** 10.3390/diagnostics11020306

**Published:** 2021-02-14

**Authors:** Ryan Tsz-Hei Tse, Hongda Zhao, Christine Yim-Ping Wong, Carol Ka-Lo Cheng, Angel Wing-Yan Kong, Qiang Peng, Peter Ka-Fung Chiu, Chi-Fai Ng, Jeremy Yuen-Chun Teoh

**Affiliations:** S.H. Ho Urology Centre, Department of Surgery, The Chinese University of Hong Kong, Hong Kong, China; ryantse@surgery.cuhk.edu.hk (R.T.-H.T.); hdzhao@surgery.cuhk.edu.hk (H.Z.); christinewong@surgery.cuhk.edu.hk (C.Y.-P.W.); carolcheng@surgery.cuhk.edu.hk (C.K.-L.C.); angel@surgery.cuhk.edu.hk (A.W.-Y.K.); pengqiang@surgery.cuhk.edu.hk (Q.P.); peterchiu@surgery.cuhk.edu.hk (P.K.-F.C.); ngcf@surgery.cuhk.edu.hk (C.-F.N.)

**Keywords:** urinary cell-free DNA, bladder cancer, detection, non-invasive, biomarker

## Abstract

Urinary bladder cancer is a common urological cancer. Although flexible cystoscopy is widely employed in bladder cancer detection, it is expensive, invasive, and uncomfortable to the patients. Recently, urinary cell-free DNA (ucfDNA) isolated from urine supernatant has been shown to have great potential in bladder cancer detection and surveillance. Molecular features, such as integrity and concentration of ucfDNA, have been shown to be useful for differentiating bladder cancer patients from healthy controls. Besides, bladder cancer also exhibits unique genetic features that can be identified from sequencing and expression of ucfDNA. Apart from bladder cancer detection, ucfDNA is also useful for molecular classification. For example, ucfDNA exhibits significant differences, both molecularly and genetically, in non-muscle-invasive and muscle-invasive bladder cancers. There is no doubt that ucfDNA is a very promising tool for future applications in the field of bladder cancer.

## 1. Introduction

Urinary bladder cancer is the ninth most common cancer worldwide [1] with approximately 550,000 new cases diagnosed and 220,000 deaths every year [2,3]. Currently, flexible cystoscopy is the gold standard for diagnosing bladder cancer. However, it is an invasive approach, and subtle malignant changes can be easily missed. A simple, inexpensive, non-invasive approach for cancer detection has to be developed, and urinary markers may serve as a useful assay for bladder cancer diagnosis and surveillance [4].

Liquid biopsy has yielded enormous interest in the field of cancer diagnostics. Among all the different types of body fluids, urine specimen, in particular, is very promising for bladder cancer as it is easily obtainable, non-invasive, and it is in direct contact with the tumor within the urinary tract [5]. Studies showed that urinary supernatant and sediments had higher rates of cumulative mutations of DNA when compared to that isolated from plasma [6]. Analysis of biomolecules in urine can be performed to identify promising biomarkers for bladder cancer detection. Biomolecules that are commonly found in urine include cellular DNA, cell-free DNA (cfDNA), different classes of RNA, proteins, and exosomes. Among them, urinary cell-free DNA (ucfDNA) reflects the genomic content of tumor cells. Several studies also reveal that ucfDNA had a higher tumoral genome than cellular DNA, which gives rise to the utilization of ucfDNA in bladder cancer detection [6]. With the advances in next-generation sequencing (NGS) of ucfDNA, specific alternation patterns can be observed, which makes cancer prediction possible. In this review, we discuss the application of ucfDNA in the detection and diagnosis of urinary bladder cancer, as wells as the limitations and future development of this non-invasive biomarker.

## 2. Urinary Cell-Free DNA Features

**Figure 1:**
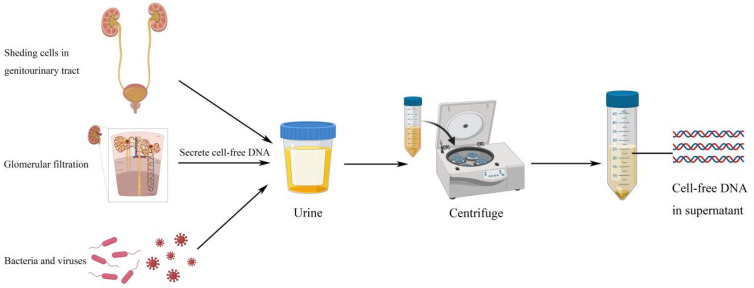
Urinary cell-free DNA can come from shedding cells in genitourinary tract, glomerular filtration process, and infecting bacteria or viruses in urogenital tract. Then, after centrifuging, researchers can get cell-free DNA in urine supernatant.

The origins of ucfDNA can be classified into three categories, namely urinary tract cells’ DNA, transrenal DNA, and non-human DNA. Urinary tract cells’ DNA originates from cells shedding from the genitourinary tract into urine, transrenal DNA comes from cfDNA in the circulation passing through glomerular filtration, and nonhuman DNA originates from bacteria and viruses infecting the urogenital tract [7,8]. According to its size, ucfDNA can be further categorized into two groups: high molecular weight and low molecular weight ucfDNA. High molecular weight ucfDNA fragments are longer than 1 kbp, and they originate mainly from necrotic cells along the urogenital tract or from lymphocytes that are normally present in the urine. Low molecular weight ucfDNA fragments usually come from the circulation or from apoptotic cells that are in contact with the urine. Depending on the type of techniques being employed, the length of low molecular weight ucfDNA varies from 10–400 bp [8]. The majority of ucfDNA originates from apoptotic or necrotic cells that are exfoliated from the urogenital system, and under normal conditions, 3 × 10^6^ epithelial cells can be shed into the urine from the urogenital tract every day [9]. Therefore, ucfDNA has a great potential in studying urological malignancies such as bladder cancer. Bladder cancer can release tumor cells into the urine, hence, it may be possible to identify urinary biomarkers for cancer detection and diagnosis, surveillance of tumor recurrence and progression, monitoring of treatment response, and predicting treatment prognosis [8]. Several parameters of ucfDNA can be employed for the detection and diagnosis of bladder cancers, for example, ucfDNA concentrations and ucfDNA integrities [4,10,11,12]. Based on the fact that apoptotic cells release highly-fragmented ucfDNA in normal conditions, this would result in small and uniform DNA fragments of 185–200 bp [13]. On the other hand, necrotic tumor cells usually release ucfDNA with maintained integrity [14]. By assessing the integrities of ucfDNA, which is defined as the ratio of longer to shorter DNA fragments, and the concentrations of ucfDNA of particular bp, clinicians can easily differentiate between patients of healthy conditions and patients with bladder cancers (Figure 1). Apart from molecular investigations of ucfDNA, studies also focus on sequencing of ucfDNA and measuring the expression levels of ucfDNA in order to identify mutated genes in bladder cancer patients. They may play an important role in early diagnosis and detection of bladder cancer [8].

## 3. Bladder Cancers Detection and Diagnosis

**Figure 2:**
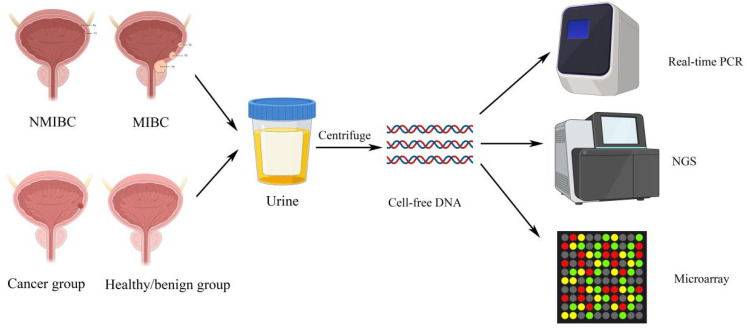
Studies reported that there existed a significant difference of the urinary cell-free DNA between bladder cancer patients and healthy or benign groups. By real-time qPCR and next-generation sequencing, we can accurately test the amount of ucfDNA, thus achieving non-invasive detection of bladder cancer. Which means we have a chance to discriminate bladder cancer patients from healthy group, even differentiating bladder cancer patients of different subtypes, thus achieving early detection of primary and recurrent patients.

Various studies analyzed the concentrations and integrities of ucfDNA as a potential non-invasive marker for bladder cancers. Several studies collected patients’ early morning urine, as it contained relatively more abundant amounts of ucfDNA, originating from the cells and cellular debris that were exfoliated from the urogenital tract during the night-time [15]. Nonetheless, Brisuda et al. observed that the second morning portions yielded higher values of ucfDNA concentrations, yet the volume was significantly lower. The second voided portion, collected two to three hours after the first micturition, was considered appropriate for downstream analysis based on two main reasons. First, by using the second voided urine, the dwell time of urine inside the bladder could be standardized. Second, the second portion of morning urine could avoid high concentration and high level of cytolysis [10]. Sequencing and expression levels of ucfDNA by next-generation sequencing are also able to detect potential genes of interest in detection of bladder cancers (Figure 2). For example, *TP53* and *FGFR3* mutations are frequent events in bladder cancers [16]. Since the bladder is in direct contact with the urine, thus detecting overexpressed or downregulated genes from exfoliated bladder tumor cells in ucfDNA that are associated with cancer progression, this can be an approach for early detection of bladder cancers (Table 1).

### 3.1. Urinary Cell-free DNA Integrity

Casadio et al. investigated the ucfDNA integrity in urine supernatant in order to assess whether it can be a potential non-invasive marker to distinguish bladder cancer patients from healthy individuals or from patients with benign urological diseases [12]. The authors analyzed ucfDNA fragments longer than 250 bp, corresponding to three oncogenes sequences: *c-Myc, HER2,* and *BCAS1*. These three oncogenes are frequently amplified in bladder cancers, mainly exhibiting their roles from pre-malignant tumor stage to primary invasive high-grade, as well as metastatic, bladder cancers [17,18,19]. Results showed that the median values of ucfDNA integrities were significantly different between non-cancerous and cancerous individuals. The authors observed that ucfDNA integrity was significantly lower in healthy individuals, and more than 40-fold higher in bladder cancer patients. The results indicated the ucfDNA in cancer patients were predominantly of long fragments, which pointed towards its origin of non-apoptotic cell, i.e., likely from necrotic cancer cells. Intriguingly, the authors observed that ucfDNA integrity had a higher sensitivity than conventional non-invasive cytology, especially for early-stage and low-grade tumors, suggesting that ucfDNA could be a promising biomarker in the early detection of bladder cancer. The authors also demonstrated that ucfDNA integrities could be a reliable indicator to differentiate cancer patients from healthy controls and symptomatic non-cancerous patients. Specificities of detecting ucfDNA at the three genome regions were similar in healthy individuals and symptomatic non-cancerous patients, indicating that false positives were rare events [12].

### 3.2. Urinary Cell-free DNA Concentrations

Another study focused on the total amount of ucfDNA in distinguishing bladder cancers of different stages from control individuals. Brisuda et al. observed a significantly higher total amount of ucfDNA in urine supernatant in bladder cancer patients [10]. Furthermore, control groups were subdivided into healthy group and benign group (patients with benign urological diseases). There was a significant difference in the total amount of ucfDNA between healthy and cancer groups. Surprisingly, no difference was observed between the benign group and cancer group. The authors further divided the cancer group into pTa group and higher-stages group (from pT1–4). A significant difference in the total amount of ucfDNA was observed between control subgroups and the higher-stages group. Although pTa group’s ucfDNA total amount showed a significant difference when compared to the advanced tumor group, no difference was noted when it was compared to the control subgroups. The above observations suggest that there could be similarities between pTa patient group and patients with benign urological disease with regards to ucfDNA levels, and a higher ucfDNA amount was expected in bladder cancer patients of higher stages and grades [10]. The authors concluded that ucfDNA could be a promising biomarker to distinguish bladder cancers of different stages.

Chang et al. also investigated the potential role of ucfDNA in urinary supernatant for bladder cancer detection [4]. As the values of urinary biochemicals are widely adjusted by urine creatinine (UCr) value and varies depending on urine volume [20,21,22], the authors determined relative ucfDNA concentrations by dividing ucfDNA by UCr concentrations (ucfDNA/UCr). In the same study, they also compared the sensitivity and specificity of two methods, PicoGreen and 400-bp amplicon, of ucfDNA quantification. Their results showed that ucfDNA/UCr were significantly higher in all bladder cancer patients when compared to controls for both PicoGreen and 400-bp methods. Intriguingly, the median concentration of 400-bp ucfDNA/UCr in bladder cancer patients were 300-fold higher than controls, whereas PicoGreen ucfDNA/UCr median concentrations only showed a 1.5-fold increase in bladder cancer patients [4]. PicoGreen ucfDNA/UCr had a positive predictive value, negative predictive value, and diagnostic accuracy of 33.3%, 70.0%, and 48.6%, respectively. On the other hand, the positive predictive value, negative predictive value, and diagnostic accuracy of 400-bp ucfDNA/UCr were 54.4%, 93.1%, and 76.0%, respectively. The observations above suggested that 400-bp ucfDNA/UCr was a better tumor marker in terms of sensitivity and specificity for detecting bladder cancer and discriminating bladder cancer patients from control individuals. Chang et al. also excluded the possibility of leukocytes and bacteria interference from urinary tract infection with ucfDNA concentrations. Leukocyte DNA, if present, did not significantly interfere with ucfDNA concentrations for both methods, indicating that ucfDNA is a reliable biomarker to detect bladder cancers [4].

Zancan et al. also performed the quantification of ucfDNA for bladder cancer detection [23]. In their study, the authors observed that all of the bladder cancer patients had ucfDNA concentrations exceeding 250 ng/mL, whereas only less than 40% of healthy individuals with negative cystoscopies had ucfDNA concentration of more than 250 ng/mL. Based on these results, the authors suggested that the ucfDNA concentration of 250 ng/mL could be taken as threshold value between a negative and a positive prediction [23].

### 3.3. Urinary Cell-free DNA Sequencing

Apart from the molecular level, Ou et al. showed that ucfDNA possessed a great diagnostic value in the genetic level. The authors discovered that ucfDNA isolated from urine supernatant yielded a powerful panel of genes for detection and diagnosis of bladder cancers. The panel of five target genes were *TERT, FGFR3, TP53, PIK3CA, and KRAS,* and all of them showed a high mutation rate in cancer patients [6]. *TERT*, *FGFR3,* and *TP53* had frequent mutations in bladder cancers (COSMIC database), and the five-gene panel held high values of detection, diagnostic, and monitoring of bladder cancers. They also demonstrated that urine of bladder cancer patients showed the highest total number of mutations in terms of both ucfDNA and cellular DNA, while healthy controls showed the least. Moreover, the area under curve of the five-gene panel from urinary supernatant (ucfDNA) was 0.94, which indicated an excellent diagnostic performance. As a result, the authors suggested that ucfDNA is a powerful non-invasive biomarker for detecting bladder cancers [6].

Cheng et al. sequenced ucfDNA to investigate its potential in bladder cancer detection. The authors employed shallow-depth paired-end genome-wide bisulfite sequencing of ucfDNA and utilized three independent parameters, methylation deconvolution, global hypomethylation, and copy number alternations (CNAs), to detect bladder cancers [24]. By sequencing ucfDNA from one T3 high-grade bladder cancer patient’s urine and tumor, they observed a high concordance in terms of hypomethylation and CNAs. The authors noted that ucfDNA fragments were contributed to by mainly bladder tumor, urothelium, and blood cells. Of note, contribution from bladder tumor was significantly higher in high-grade and muscle-invasive patients. The genome-wide bisulfite sequencing results also revealed that ucfDNA from bladder cancer cases showed significant hypomethylation and CNAs across the whole genome. To further prove that these three parameters were useful in detecting bladder cancer, the authors compared four postoperative urine with preoperative one. All urine samples showed different extents of reduction in the levels of the three parameters, and three of them reverted to levels below the threshold as defined by cancer-free controls. Although each of the three parameters could differentiate bladder cancers from healthy individuals, their individual sensitivity was relatively low for low-grade NMIBC. However, by combining the three approaches altogether, an overall sensitivity of 93.5% could be achieved. For urine cytology, only 4 out of 42 tested positive within a 6-month period prior to urologic surgeries. The authors further applied this concept in detecting recurrence, progression, and monitoring tumor load in response to different therapeutic strategies, making ucfDNA a useful tool for evaluating disease severity and monitoring treatment response [24].

Birkenkamp-Demtröder et al. performed a retrospective pilot study of twelve patients to investigate the utilization of ucfDNA in the surveillance of recurrent or progressive/metastatic disease. The authors isolated cfDNA from urine and plasma supernatant, identified tumor-specific genomic variants from next-generation sequencing, and aimed to develop and apply personalized assays for cancer surveillance [25]. In brief, three methods were employed by the authors to establish personalized assays, whole genome sequencing, whole exome sequencing, and mate-pair sequencing of DNA from normal tissue and bladder tumor, followed by identification of genomic structural variants. After validation of the genomic variants by PCR, Sanger sequencing was employed to determine tumor-specific breakpoint at base-pair resolution to detect tumor-specific ucfDNA from urine samples. The patients were divided into two groups, namely the progressive (PRO) group and the recurrent (REC) group. PRO group was defined as NMIBC which progressed to muscle-invasive or metastatic disease, and the REC group was defined as recurrent NMIBC. Their results showed that tumor-specific ucfDNA was detected in 96.5% of the PRO group, and only 50% in the REC group. High levels of tumor-specific ucfDNA were shown in all PRO group patients. Of note, 83% of them had tumor-specific ucf DNA detected several months before clinical progression to muscle-invasive disease, even when there was very low or undetectable cfDNA in plasma; this suggested that ucfDNA might be more sensitive than plasma in bladder cancer detection [25]. Interestingly, the authors found out that one patient from the PRO group exhibited five genomic variants at initial visit before his muscle-invasive tumor resected 4.5 years later. Besides, another patient from the PRO group possessed high levels of tumor-specific ucfDNA at the diagnosis of NMIBC, and with increasing levels over time. Moreover, two patients from the REC group with high levels of tumor-specific ucfDNA reverted to undetectable levels after bacillus Calmette–Guérin instillation. From the above three observations, authors suggested that routine ucfDNA analysis could be a good supplement to conventional cystoscopy and as a tool for early detection of progression and disease surveillance. As tumor-specific ucfDNA was significantly higher in invasive/metastatic patients than in recurrent patients, the authors suggested that ucfDNA could reflect disease invasiveness instead of merely the presence of tumors within the urinary bladder. In summary, tumor-specific ucfDNA detected by personalized assays could identify tumor evolution, aggressiveness, and invasiveness; early detection of progression and metastasis is possible. The authors suggested this method could be applied in the monitoring of treatment responses, and personalized therapy strategies could be customized [25].

### 3.4. Urinary Cell-free DNA Expression

NMIBC has a better prognosis than MIBC and metastatic bladder cancers. However, without early detection and treatment, NMIBC may progress to MIBC and metastatic diseases [26]. We may argue the detection of NMIBC is perhaps more important than the detection of advanced diseases. Kim et al. investigated the value of Topoisomerase-II alpha (*TopoIIA*) in ucfDNA in differentiating NMIBC from MIBC. *TopoIIA* is an isoform of DNA gyrase that plays an important role in cell cycle, and an increased expression of *TopoIIA* was reported to be associated with higher recurrence rate of NMIBC [27]. It was found that *TopoIIA* is highly expressed in bladder cancer tumors, and expression of urinary *TopoIIA* cell-free DNA amplified by PCR was significantly higher in cancerous patients than in healthy controls or hematuria patients. Interestingly, the expression levels were significantly higher in MIBC patients than in NMIBC patients, suggesting that high levels of *TopoIIA* amplified from ucfDNA were associated with more aggressive pathologies. The authors also demonstrated a high sensitivity of more than 70%, reflecting that *TopoIIA* ucfDNA could be a potential marker in detecting bladder cancers [28].

Around 15% to 61% of NMIBC patients developed recurrence at 1 year, and even up to 31% to 78% at 5 years [29]. Being able to predict cancer recurrence and progression are very important to guide adjunct treatment strategies. Xu et al. developed a novel non-invasive diagnostic marker for NMIBC. The authors measured the ratios of IQ motif-containing GTPase activating protein 3 (*IQGAP3*)/bone morphogenetic protein 4 (*BMP4*) and *IQGAP3*/family with sequence similarity 107 (*FAM107A*) in ucfDNA. Previous studies suggested that *IQGAP3* was overexpressed at mRNA level in multiple cancer tissues, including bladder cancer. *BMP4* was shown to be downregulated in bladder cancer, where better progression-free survival (PFS) was observed in advanced ovarian cancer patients with strong expression of *BMP-4* [30,31,32]. Their results showed that *IQGAP3/BMP4* overexpression was observed in higher-stage and higher-grade diseases with high risks of recurrence and progression, whereas *IQGAP3/FAM107A* overexpression was associated with larger tumor size and disease progression. High *IQGAP3/BMP4* ratio was significantly associated with worse recurrence-free survival (RFS); high *IQGAP3/BMP4* and high *IQGAP3/FAM107A* ratios were both significantly associated with worse PFS [33]. The authors also found that MIBC patients had significantly higher ratios than NMIBC patients, suggesting that these ucfDNA biomarkers were associated with disease recurrence and progression. As the measurement of two expressed genes were performed, the authors suggested that internal controls were not necessary. As a result, the ratios might be an accurate predictive biomarker in ucfDNA for bladder cancer detection and prognosis with maximum discriminatory ability [33].

## 4. Limitations

Utilization of ucfDNA as a biomarker in bladder cancer detection has been shown to be accurate in various studies, and it has a very promising prospect for translating into clinical practice. Nevertheless, there are severe limitations that should not be neglected. First of all, a standard method of ucfDNA quantification and amplification has to be developed, as different techniques may have varied sensitivity and specificity, resulting in an unacceptable high rate of false positives, which will affect the results of downstream analysis [11]. Besides, several studies showed that the parameters in ucfDNA exhibited lower sensitivity in detecting bladder cancers of lower stages and grades, such as pTa patients. This can be a big problem as this subgroup represents a large proportion among all bladder cancer patients. Hence, the sensitivity of ucfDNA for this subgroup must be enhanced in order to increase its practicality in the future.

Secondly, the exact mechanism and the origins of different ucfDNA fragments should be further investigated. Besides tumor necrosis, it was suggested that short fragments of ucfDNA may also exist due to time-dependent fragmentation [34]. Nontumor cfDNA could be shed into the urine from systemic circulation; a longer degradation time could result in a high amount of short-fragment ucfDNA [24]. Ongoing studies on the size and fragmentation of ucfDNA, as well as the background DNA from normal tissues, should be carried out in order to optimize the ability and accuracy of utilizing this non-invasive marker in bladder cancer detection. Understanding the nature of such process may also be useful for extending the use of urinary biomarkers for patients with upper tract urothelial carcinomas.

Thirdly, levels of ucfDNA may be altered by various parameters and conditions, such as the number and size of tumors (tumor surface), grades, stages, presence of urinary tract infection, and leukocyturia. It is difficult to have an accurate measurement of all these factors, and it is therefore difficult to adjust. Besides, sources other than urinary bladder may also contribute to the total amount of ucfDNA, such as different cells of multifocal and polyclonal tumors. Cells of a heterogenous tumor may also exfoliate into urine which may alter the concentration of ucfDNA [35]. Moreover, ucfDNA concentrations fluctuate not only in cancerous situations, but also in some benign urological diseases [10], therefore, the results of ucfDNA in symptomatic individuals should be verified.

Fourthly, most studies in ucfDNA for bladder cancer detection were limited by small sample sizes. Therefore, external validation becomes particularly important. Ongoing recruitment of bladder cancer patients with long-term follow up is crucial [10,11,12,23,33]. A better understanding of these biomarkers with stratification to different stages of bladder cancer and its relation to high-risk features such as carcinoma-in-situ is warranted.

Last but not least, the evidence on the utility of ucfDNA for guiding subsequent treatment choices and predicting prognosis is very limited [33]. Future development of ucfDNA as a reliable pretreatment biomarker is especially important as novel agents, such as immune check point inhibitors, come into place.

## 5. Conclusions

Bladder cancer is a common urological cancer worldwide, and flexible cystoscopy is commonly performed for bladder cancer detection. However, flexible cystoscopy is expensive and invasive, making it not a very attractive diagnostic method. Urine as a liquid biopsy has gained interest in recent years. Several biomolecules are present in urine, and among them, ucfDNA in urine supernatant has great potential in bladder cancer detection. Various studies have studied the integrity, concentration, sequencing, and expression results of ucfDNA in relation to bladder cancer diagnosis. There is compelling evidence that ucfDNA could differentiate cancerous patients from healthy individuals, and, in addition to this, it could differentiate bladder cancer patients of different stages. Therefore, ucfDNA is a very promising tool that has a great potential to translate into clinical practice. Future investigations on ucfDNA as a prognostic marker and a predictive marker for guiding subsequent treatment are warranted.

## Figures and Tables

**Table 1:** Summary of different parameters and techniques and potential markers in urinary cell-free DNA (ucfDNA) for bladder cancer detection, monitoring, and surveillance.

Parameters/Techniques	Markers	Descriptions
ucfDNA integrity	Long ucfDNA fragments (>250 bp)	Long fragmented ucfDNA, originated from necrotic tumor cells, were significantly more abundant in bladder cancer patients than in healthy individuals and symptomatic non-cancerous patients.
ucfDNA concentration	Total amount of ucfDNA	Significantly higher total amount of ucfDNA in bladder cancer patients than in healthy and benign patients. Total amount of ucfDNA was higher in patients with higher grades and stages of bladder cancer.
	400-bp ucfDNA/urine creatinine (Ucr) and PicoGreen ucfDNA/UCr	ucfDNA isolated from both methods were significantly higher in bladder cancer patients. 400-bp ucfDNA/UCr was more sensitive and specific than PicoGreen ucfDNA/UCr.
	ucfDNA 250 ng/mL concentration threshold	All cancer patients with concentrations of ucfDNA exceeded this threshold, while only less than half of the healthy controls did.
ucfDNA sequencing	*TERT*, *FGFR3*, *TP53*, *PIK3CA* and *KRAS* 5-genes panel	The 5-gene panel was generated from frequently mutated genes in bladder cancers. Highest total number of mutations were observed in bladder cancer patients. It had a high sensitivity in detection and monitoring of bladder cancers, and this panel had an area under curve (AUC) of 0.94.
	Methylation deconvolution, global methylation and copy number alterations (CNAs)	High concordance between tumor and ucfDNA in terms of hypomethylation and CNAs. Bladder cancer patients exhibited significantly hypomethylation and CNAs. Post-operative patients showed lower levels of all three parameters. Sensitivity and specificity were higher when all three parameters were combined.
	Personalized assay	Tumor-specific ucfDNA isolated from personalized assay were higher in progressive/metastatic disease than in recurrent non-muscle invasive bladder cancer (NMIBC). Genomic variants in ucfDNA could be detected prior to tumor resection. Chemotherapy could affect levels of tumor-specific ucfDNA.
ucfDNA Expression	*TopoIIA* ucfDNA	*TopoIIA* ucfDNA expression level was significantly higher in bladder cancer patients than inhealthy individuals, and higher in muscle invasive bladder cancer (MIBC) than in NMIBC.
	*IQGAP3/BMP4* and *IQGAP3/FAM107A*	*IQGAP3/BMP4* was overexpressed in high grade and stage bladder cancers, *IQGAP3/FAM107A* was overexpressed in larger tumor size and progression. Both higher ratios were associated with worse progression-free survival (PFS), and a high *IQGAP3/BMP4* ratio was also associated with worse recurrence-free survival (RFS).

## Data Availability

All available data is presented in the manuscript.

## References

[1] Ferlay J., Soerjomataram I., Dikshit R., Eser S., Mathers C., Rebelo M., Parkin D.M., Forman D., Bray F. (2015). Cancer incidence and mortality worldwide: Sources, methods and major patterns in GLOBOCAN 2012. Int. J. Cancer.

[2] Bray F., Ferlay J., Soerjomataram I., Siegel R.L., Torre L.A., Jemal A. (2018). Global cancer statistics 2018: GLOBOCAN estimates of incidence and mortality worldwide for 36 cancers in 185 countries. CA Cancer J. Clin..

[3] Teoh J.Y., Huang J., Ko W.Y., Lok V., Choi P., Ng C.F., Sengupta S., Mostafid H., Kamat A.M., Black P.C. (2020). Global Trends of Bladder Cancer Incidence and Mortality, and Their Associations with Tobacco Use and Gross Domestic Product Per Capita. Eur. Urol..

[4] Chang H.W., Tsui K.H., Shen L.C., Huang H.W., Wang S.N., Chang P.L. (2007). Urinary cell-free DNA as a potential tumor marker for bladder cancer. Int. J. Biol. Markers.

[5] Ward D.G., Bryan R.T. (2017). Liquid biopsies for bladder cancer. Transl. Androl. Urol..

[6] Ou Z., Li K., Yang T., Dai Y., Chandra M., Ning J., Wang Y., Xu R., Gao T., Xie Y. (2020). Detection of bladder cancer using urinary cell-free DNA and cellular DNA. Clin. Transl. Med..

[7] Salvi S., Casadio V. (2019). Urinary Cell-Free DNA: Potential and Applications. Methods Mol. Biol..

[8] Lu T., Li J. (2017). Clinical applications of urinary cell-free DNA in cancer: Current insights and promising future. Am. J. Cancer Res..

[9] Bryzgunova O.E., Laktionov P.P. (2015). Extracellular Nucleic Acids in Urine: Sources, Structure, Diagnostic Potential. Acta Nat..

[10] Brisuda A., Pazourkova E., Soukup V., Horinek A., Hrbacek J., Capoun O., Svobodova I., Pospisilova S., Korabecna M., Mares J. (2016). Urinary Cell-Free DNA Quantification as Non-Invasive Biomarker in Patients with Bladder Cancer. Urol. Int..

[11] Zancan M., Galdi F., Di Tonno F., Mazzariol C., Orlando C., Malentacchi F., Agostini M., Maran M., Del Bianco P., Fabricio A.S. (2009). Evaluation of cell-free DNA in urine as a marker for bladder cancer diagnosis. Int. J. Biol. Markers.

[12] Casadio V., Calistri D., Tebaldi M., Bravaccini S., Gunelli R., Martorana G., Bertaccini A., Serra L., Scarpi E., Amadori D. (2013). Urine cell-free DNA integrity as a marker for early bladder cancer diagnosis: Preliminary data. Urol. Oncol..

[13] Sozzi G., Conte D., Leon M., Ciricione R., Roz L., Ratcliffe C., Roz E., Cirenei N., Bellomi M., Pelosi G. (2003). Quantification of free circulating DNA as a diagnostic marker in lung cancer. J. Clin. Oncol..

[14] Jahr S., Hentze H., Englisch S., Hardt D., Fackelmayer F.O., Hesch R.D., Knippers R. (2001). DNA fragments in the blood plasma of cancer patients: Quantitations and evidence for their origin from apoptotic and necrotic cells. Cancer Res..

[15] Casadio V., Salvi S. (2019). Urinary Cell-Free DNA: Isolation, Quantification, and Quality Assessment. Methods Mol. Biol..

[16] Cancer Genome Atlas Research Network (2014). Comprehensive molecular characterization of urothelial bladder carcinoma. Nature.

[17] Tabach Y., Kogan-Sakin I., Buganim Y., Solomon H., Goldfinger N., Hovland R., Ke X.S., Oyan A.M., Kalland K.H., Rotter V. (2011). Amplification of the 20q chromosomal arm occurs early in tumorigenic transformation and may initiate cancer. PLoS ONE.

[18] Nord H., Segersten U., Sandgren J., Wester K., Busch C., Menzel U., Komorowski J., Dumanski J.P., Malmstrom P.U., Diaz de Stahl T. (2010). Focal amplifications are associated with high grade and recurrences in stage Ta bladder carcinoma. Int. J. Cancer.

[19] Hansel D.E., Swain E., Dreicer R., Tubbs R.R. (2008). HER2 overexpression and amplification in urothelial carcinoma of the bladder is associated with MYC coamplification in a subset of cases. Am. J. Clin. Pathol..

[20] Erhola M., Toyokuni S., Okada K., Tanaka T., Hiai H., Ochi H., Uchida K., Osawa T., Nieminen M.M., Alho H. (1997). Biomarker evidence of DNA oxidation in lung cancer patients: Association of urinary 8-hydroxy-2’-deoxyguanosine excretion with radiotherapy, chemotherapy, and response to treatment. FEBS Lett..

[21] Zhang J., Tong K.L., Li P.K., Chan A.Y., Yeung C.K., Pang C.C., Wong T.Y., Lee K.C., Lo Y.M. (1999). Presence of donor- and recipient-derived DNA in cell-free urine samples of renal transplantation recipients: Urinary DNA chimerism. Clin. Chem..

[22] Dyer A.R., Greenland P., Elliott P., Daviglus M.L., Claeys G., Kesteloot H., Ueshima H., Stamler J., Group I.R. (2004). Evaluation of measures of urinary albumin excretion in epidemiologic studies. Am. J. Epidemiol..

[23] Zancan M., Franceschini R., Mimmo C., Vianello M., Di Tonno F., Mazzariol C., Malossini G., Gion M. (2005). Free DNA in urine: A new marker for bladder cancer? Preliminary data. Int. J. Biol. Markers.

[24] Cheng T.H.T., Jiang P., Teoh J.Y.C., Heung M.M.S., Tam J.C.W., Sun X., Lee W.S., Ni M., Chan R.C.K., Ng C.F. (2019). Noninvasive Detection of Bladder Cancer by Shallow-Depth Genome-Wide Bisulfite Sequencing of Urinary Cell-Free DNA for Methylation and Copy Number Profiling. Clin. Chem..

[25] Birkenkamp-Demtroder K., Nordentoft I., Christensen E., Hoyer S., Reinert T., Vang S., Borre M., Agerbaek M., Jensen J.B., Orntoft T.F. (2016). Genomic Alterations in Liquid Biopsies from Patients with Bladder Cancer. Eur. Urol..

[26] Sylvester R.J., van der Meijden A.P., Oosterlinck W., Witjes J.A., Bouffioux C., Denis L., Newling D.W., Kurth K. (2006). Predicting recurrence and progression in individual patients with stage Ta T1 bladder cancer using EORTC risk tables: A combined analysis of 2596 patients from seven EORTC trials. Eur. Urol..

[27] Ohashi Y., Sasano H., Yamaki H., Shizawa S., Kikuchi A., Shineha R., Akaishi T., Satomi S., Nagura H. (1999). Topoisomerase II alpha expression in esophageal squamous cell carcinoma. Anticancer Res..

[28] Kim Y.H., Yan C., Lee I.S., Piao X.M., Byun Y.J., Jeong P., Kim W.T., Yun S.J., Kim W.J. (2016). Value of urinary topoisomerase-IIA cell-free DNA for diagnosis of bladder cancer. Investig. Clin. Urol..

[29] Dwivedi U.S., Kumar A., Das S.K., Trivedi S., Kumar M., Sunder S., Singh P.B. (2009). Relook TURBT in superficial bladder cancer: Its importance and its correlation with the tumor ploidy. Urol. Oncol..

[30] Kuzaka B., Janiak M., Wlodarski K.H., Radziszewski P., Wlodarski P.K. (2015). Expression of bone morphogenetic protein-2 and -7 in urinary bladder cancer predicts time to tumor recurrence. Arch. Med. Sci..

[31] Laatio L., Myllynen P., Serpi R., Rysa J., Ilves M., Lappi-Blanco E., Ruskoaho H., Vahakangas K., Puistola U. (2011). BMP-4 expression has prognostic significance in advanced serous ovarian carcinoma and is affected by cisplatin in OVCAR-3 cells. Tumour Biol..

[32] Kumar D., Hassan M.K., Pattnaik N., Mohapatra N., Dixit M. (2017). Reduced expression of IQGAP2 and higher expression of IQGAP3 correlates with poor prognosis in cancers. PLoS ONE.

[33] Xu Y., Kim Y.H., Jeong P., Piao X.M., Byun Y.J., Seo S.P., Kang H.W., Kim W.T., Lee J.Y., Ryu D.H. (2019). Urinary Cell-Free DNA IQGAP3/BMP4 Ratio as a Prognostic Marker for Non-Muscle-Invasive Bladder Cancer. Clin. Genitourin. Cancer.

[34] Cheng T.H., Jiang P., Tam J.C., Sun X., Lee W.S., Yu S.C., Teoh J.Y., Chiu P.K., Ng C.F., Chow K.M. (2017). Genomewide bisulfite sequencing reveals the origin and time-dependent fragmentation of urinary cfDNA. Clin. Biochem..

[35] Utting M., Werner W., Dahse R., Schubert J., Junker K. (2002). Microsatellite analysis of free tumor DNA in urine, serum, and plasma of patients: A minimally invasive method for the detection of bladder cancer. Clin. Cancer Res..

